# CD4 T Cells Acquire Innate Capability Upon Classical T Cell Activation

**DOI:** 10.1002/eji.70054

**Published:** 2025-09-09

**Authors:** Nima Yassini, Eva Goljat, Camilla Panetti, Matthias Rath, Nicole Joller

**Affiliations:** ^1^ Department of Quantitative Biomedicine University of Zurich Zurich Switzerland; ^2^ Center for Human Immunology University of Zurich Zurich Switzerland

**Keywords:** host/pathogens interactions, innate immunity, T helper cells

## Abstract

Memory T cells, a sizable compartment of the mature immune system, enable enhanced responses upon re‐infection with the same pathogen. We have recently shown that virus‐experienced innate acting T (T_IA_) cells can modulate infectious or autoimmune diseases through TCR‐independent IFN‐γ production. However, how these cells arise remains unclear. Here, we show that CD4 T_IA_ cells are present in various disease settings hinting towards a disease‐agnostic nature. TCR stimulation and CD28 co‐stimulation are sufficient to induce naïve murine and human CD4 T cells to become capable of cytokine‐mediated, TCR‐independent IFN‐γ responses. In true T_IA_ fashion, adoptive transfer of in vitro‐induced T_IA_ cells in mice yielded a TCR‐independent IFN‐γ response during the innate phase of a *Legionella pneumophila* infection. Our data thus shows that CD4 T_IA_ cells are more ubiquitous than anticipated and could therefore be involved in more settings than expected.

## Graphical abstract

The innate actions of the immune system allow for a rapid response to potential threats. Here we show that upon classical T cell activation, both murine and human adaptive immune cells can acquire innate capabilities allowing them to respond to cytokines in a TCR independent manner.

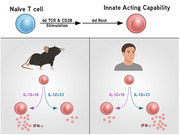

## 1 Introduction

Immunology research utilizing murine models typically uses specific‐pathogen‐free (SPF) mice to study various diseases where the immune system is or could be involved. This use of SPF mice has many benefits, most notably a controlled environment leading to reduced variability stemming from confounding variables and thus increased reproducibility of experimental findings. While this isolated study allows for a better mechanistic understanding of diseases, it neglects potential interactions originating from past pathogenic encounters that influence the immune composition which in turn could respond differently to subsequent heterologous challenges. This becomes especially apparent when considering the fact that in humans already at the age of 2 years, memory T cells constitute the vast majority of T cells at mucosal sites [1]. It is therefore important to also consider how past immune challenges could influence susceptibility to unrelated diseases.

We have previously shown that memory CD4 T cells generated during an acute lymphocytic choriomeningitis virus (LCMV) infection can be rapidly recruited and respond to an unrelated bacterial infection, such as *Legionella pneumophila*. Within 2 days, these cells produce IFN‐γ through a TCR‐independent mechanism, leading to a faster clearance of the pathogen [2]. This innate response could be evoked through IL‐12 and IL‐18 stimulation, a cytokine combination that has been shown to synergize and stimulate IFN‐γ production in T cells [3, 4, 5, 6], and to a lesser extent IL‐12 and IL‐33 stimulation [2]. Similar reports of human CD4 T cells have shown that they too can be activated through cytokines to produce IFN‐γ [7, 8, 9].

While the murine innate acting T (T_IA_) cells were beneficial in a bacterial challenge, CD4 T_IA_ cells were detrimental to the host in an autoimmune setting as they promoted an earlier onset of experimental autoimmune encephalomyelitis (EAE) [2], an autoimmune model for multiple sclerosis. Such TCR‐independent CD4 T cell‐responses have also been hinted to play a role in patients suffering from autoimmunity such as rheumatoid arthritis patients [8].

These findings suggest the involvement of CD4 T_IA_ cells in various disease settings. While we have observed the presence of CD4 T_IA_ cells during memory time points in LCMV‐ as well as vaccinia virus‐experienced mice [2], it remains unclear how CD4 T_IA_ cells are generated. In this study, we demonstrate that classical T cell activation alone is sufficient for both murine and human naïve CD4 T cells to gain cytokine responsiveness. We further show that, in contrast to murine CD4 T cells, human CD4 T cells are more responsive to IL‐33 than IL‐18 in driving cytokine‐mediated, TCR‐independent IFN‐γ responses. Finally, in vitro generated CD4 T_IA_ cells were capable of responding to a *L. pneumophila* challenge in a TCR‐independent manner, similar to in vivo generated T_IA_ cells.

## 2 Results

### 2.1 Innate Acting T Cells Are Present in Diverse Contexts

**FIGURE 1:**
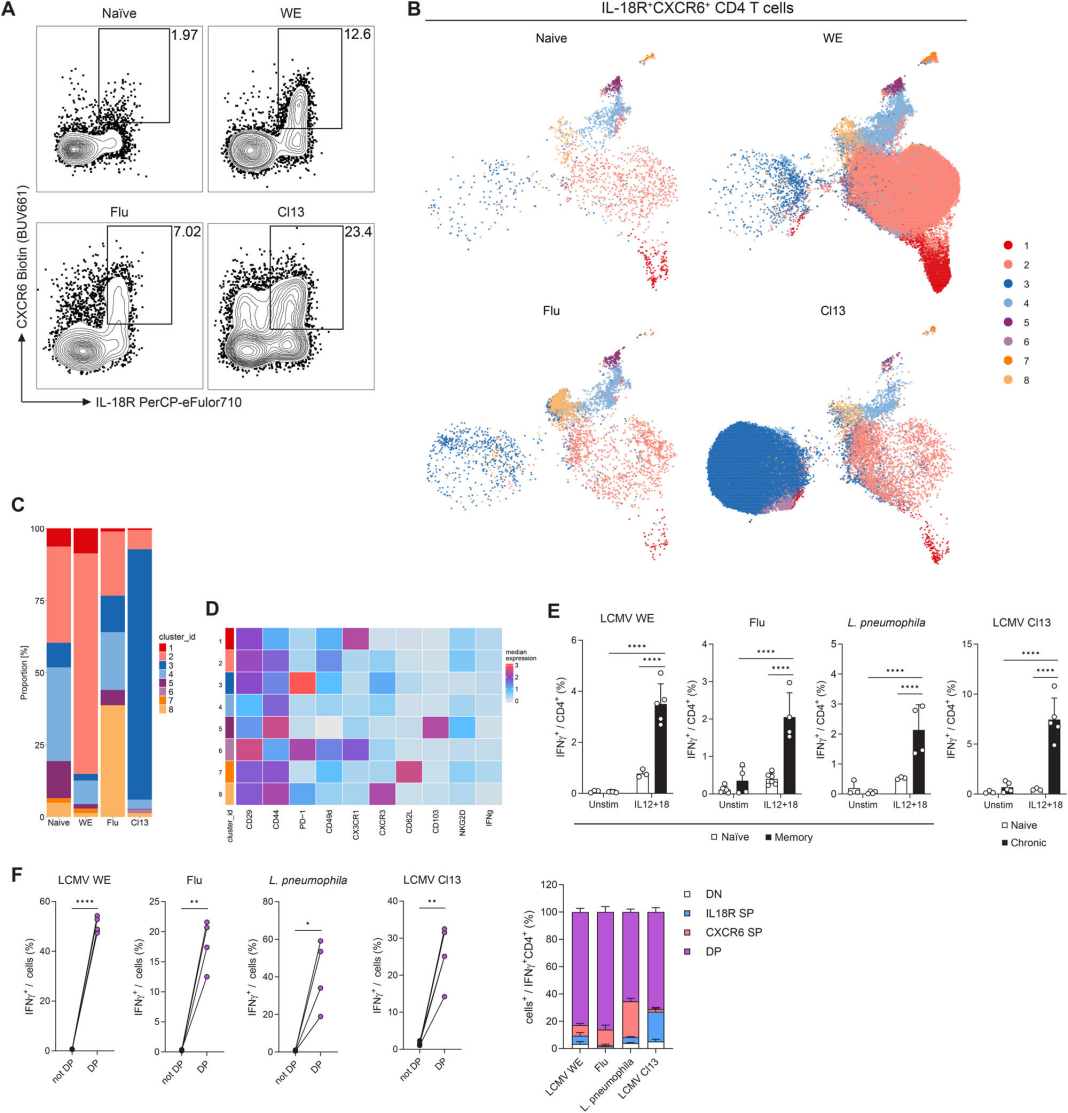
CD4 T_IA_ cells found after various infections. (A–D) Mice were infected with LCMV WE (i.v.), LCMV Cl13 (i.v.), influenza A virus (Flu, i.n.) or left naïve and immune cells of the lung were isolated from LCMV WE or Flu infected mice (day ≥ 40) or LCMV Cl13 infected mice (day ≥ 25). (A) Representative FACS plots gated on CD4 T cells. (B–D) IL‐18R^+^CXCR6^+^ CD4 T cells, gated as shown in (A), were further analyzed using unsupervised clustering (*n* = 2–5, two independent experiments). (B) UMAP, (C) proportion of each cluster per condition and (D) heatmap showing marker expression within these clusters. (E and F) Splenic CD4 T cells of LCMV WE, *L. pneumophila*, and LCMV Cl13 experienced mice and lung CD4 T cells of Flu‐experienced mice were isolated and stimulated overnight with cytokines or left untreated (*n* = 3–6, two independent experiments). (E) IFN‐γ response among CD4 T cells (two‐way ANOVA with Šídák). (F) left: IFN‐γ response among IL‐18R^+^CXCR6^+^ (DP) cells and non‐DP cells (paired *t*‐test), right: IL‐18R and CXCR6 expression among IFN‐γ^+^ CD4 T cells (DN: double negative, SP: single positive, DP: double positive).

To gain insight into how CD4 innate acting T (T_IA_) cells are generated, we first examined in which settings CD4 T_IA_ cells can be found. To this end, mice were infected with influenza A virus or the acute WE or chronic Clone 13 (Cl13) strain of LCMV. At least 40 days after the acute infections (WE memory or Flu memory) or 25 days post the chronic infection (Cl13 chronic), we examined the CD4 T cells found in the lung of these mice. Looking at the CD4 T_IA_ markers IL‐18R and CXCR6, we could clearly observe an increased expression and altered distribution when assessed by unsupervised clustering compared to naïve controls (Figure 1A, Figure S1). We then further analyzed this IL‐18R^+^CXCR6^+^ population using unsupervised clustering and observed a dominant Cluster 2 with higher VLA‐4 expression in WE memory, as we have previously shown [2], while Flu memory mice featured a prominent Cluster 8 marked by higher CXCR3 expression, and Cl13 chronically infected mice displayed a dominant Cluster 3 with high PD‐1 expression (Figure 1B‐D). Nevertheless, all clusters were represented in each of the infectious settings, suggesting that CD4 T_IA_ cells capable of responding to cytokine stimulation alone, could be generated in diverse infectious settings, although with alterations in terms of proportion and expression of additional markers.

We therefore asked if these cells, despite their differences in marker expression (VLA‐4, CXCR3, PD‐1), indeed possess the innate capability to mount an IFN‐γ response upon cytokine stimulation. To this end, we isolated CD4 T cells from mice that had undergone and cleared infection with LCMV WE, influenza A, or *L. pneumophila* or were chronically infected with LCMV Cl13. CD4 T cells were then stimulated with IL‐12 and IL‐18, which have been shown to be relevant for the T_IA_ response [2]. As previously seen, LCMV WE memory CD4 T cells showed a significantly higher IFN‐γ production compared to the naïve controls (Figure 1E). Besides this acute systemic viral infection, the acute local lung infection with influenza, the acute systemic bacterial *L. pneumophila* infection, and even the chronic systemic LCMV Cl13 infection all induced a CD4 T cell population equipped with enhanced responsiveness to cytokine stimulation. Further examination of the IL‐18R and CXCR6 expression revealed a highly enriched response in the population co‐expressing these markers in all conditions, further corroborating the utility of these markers to identify CD4 T_IA_ cells (Figure 1F).

Taken together, these results suggest that the innate acting capability of Th1 cells could be more ubiquitous than anticipated, as their responsiveness to cytokine stimulation alone could be detected with diverse infections.

### 2.2 Human CD4 T_IA_ Cells Are More Responsive to IL‐33 Than IL‐18

**FIGURE 2:**
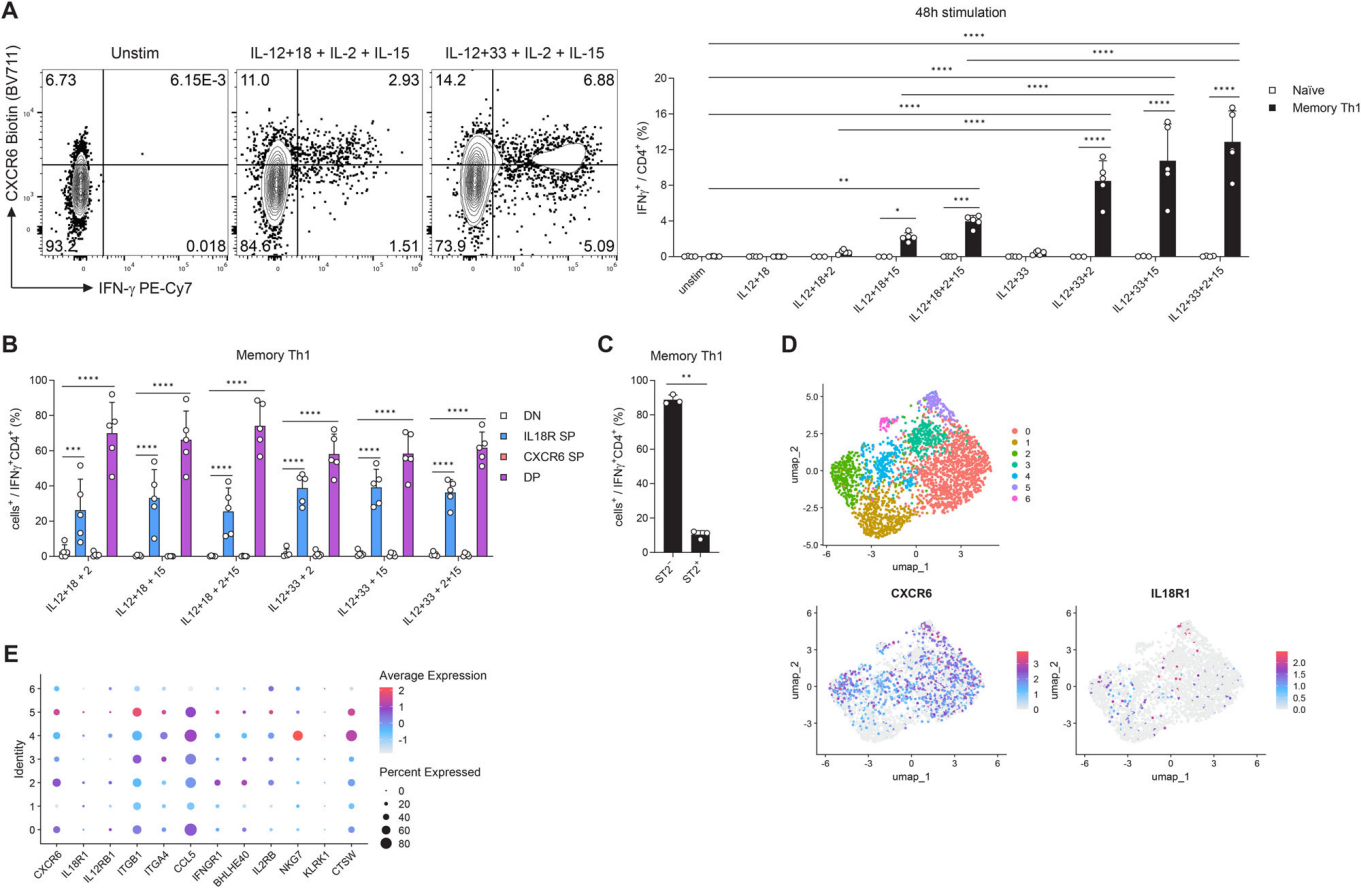
Human CD4 T cells respond strongly to IL‐12, IL‐33, IL‐2, and/or IL‐15. (A and B) Human CD4 T cells isolated from PBMCs of healthy donors were sorted for CD45RA^+^CD45RO^−^ (Naïve) or CD45RA^−^CD45RO^+^CXCR3^+^ (Memory Th1) and stimulated with the indicated cytokines (*n* = 4–5, three independent experiments). (A) The resulting IFN‐γ response is shown in representative FACS plots (left, gated on CD4 T cells) and summary graphs (right; two‐way ANOVA with Šídák). (B) Frequency of marker^+^ populations among IFN‐γ^+^ CD4 T cells (two‐way ANOVA with Dunnett). Memory Th1 cells isolated as described in (A) were incubated with IL‐12, IL‐33, IL‐2, and IL‐15 for 48 h and ST2 expression among IFN‐γ^+^ CD4 T cells was analyzed (*n* = 3, two independent experiments, paired *t*‐test). (D and E) Single cell RNA‐seq dataset of human lung CD4 T cells [10] analyzed for T_IA_ marker expression displayed as (D) UMAP or (E) Dotplot.

Because of the diverse settings from which CD4 T_IA_ cells can be generated, we next examined human CD4 T cells for their TCR‐independent cytokine responsiveness. Previous reports have shown that IL‐12 and IL‐18 in combination with some common gamma chain cytokines can elicit an IFN‐γ response in human CD4 T cells [7, 8, 9]. We have previously demonstrated that IL‐12 and IL‐33 can also elicit an IFN‐γ response in murine CD4 T cells, although to a lesser extent than IL‐12 and IL‐18 [2]. However, whether IL‐33 can activate human CD4 T cells, has not been explored. To this end, we isolated CD4 T cells from PBMCs of healthy donors, sorted CD45RA^+^CD45RO^−^ CD4 T cells (naïve) and CD45RA^−^CD45RO^+^CXCR3^+^ CD4 T cells (memory Th1) and stimulated them with combinations of IL‐12 plus IL‐18 or IL‐33, in the presence or absence of IL‐2 and IL‐15 (Figure 2A). As previously reported [8], the combination of IL‐12 and IL‐18 together with IL‐15 or with IL‐2 and IL‐15 was able to evoke an IFN‐γ response. To our surprise, the combination of IL‐12 and IL‐33 together with IL‐2 and/or IL‐15 lead to an IFN‐γ response that was more than twice as strong as that induced by IL‐18.

Looking at the expression of IL‐18R and CXCR6, a combination of receptors previously identified as an effective marker combination for murine CD4 T_IA_ cells [2], we observed that most of the IFN‐γ producing cells co‐expressed IL‐18R and CXCR6 (Figure 2B), similar to the murine setting. However, a significant portion of the IFN‐γ producing human cells was also single‐positive for IL‐18R, suggesting that for human CD4 T cells IL‐18R expression alone serves as a better marker for potential CD4 T_IA_ cells. Due to the higher potential of IL‐33 to elicit an IFN‐γ response in memory Th1 cells, we explored whether the IL‐33 receptor ST2 could capture the IFN‐γ−producing cells. However, the majority of IFN‐γ^+^ CD4 T cells were negative for the expression of ST2 (Figure 2C). These differences between murine and human CD4 T_IA_ cells prompted us to analyze expression of T_IA_ signature genes (derived from their murine transcriptional profile [2]) in a dataset of human CD4 T cells from healthy lung tissues [10] (Figure 2D,E). While some CD4 T_IA_ genes were barely detectable or expressed to a lower extent in human CD4 T cells (*IL18R1, IL12RB1*, *ITGA4*, *IFNGR1*, *BHLHE40*, *IL2RB*, *KLRK1*), others (*CXCR6*, *ITGB1*, *CCL5*, *NKG7*, *CTSW*) were expressed to a greater extent. Together, these findings show that, while functional CD4 T_IA_ cells are present in humans, these cells phenotypically differ from their murine counterpart. Most notably, human CD4 T_IA_ cells show a significantly greater responsiveness to IL‐12 plus IL‐2 and/or IL‐15 when combined with IL‐33 compared to IL‐18.

### 2.3 In Vitro‐Generated CD4 T_IA_ Cells Are Functional In Vivo

**FIGURE 3:**
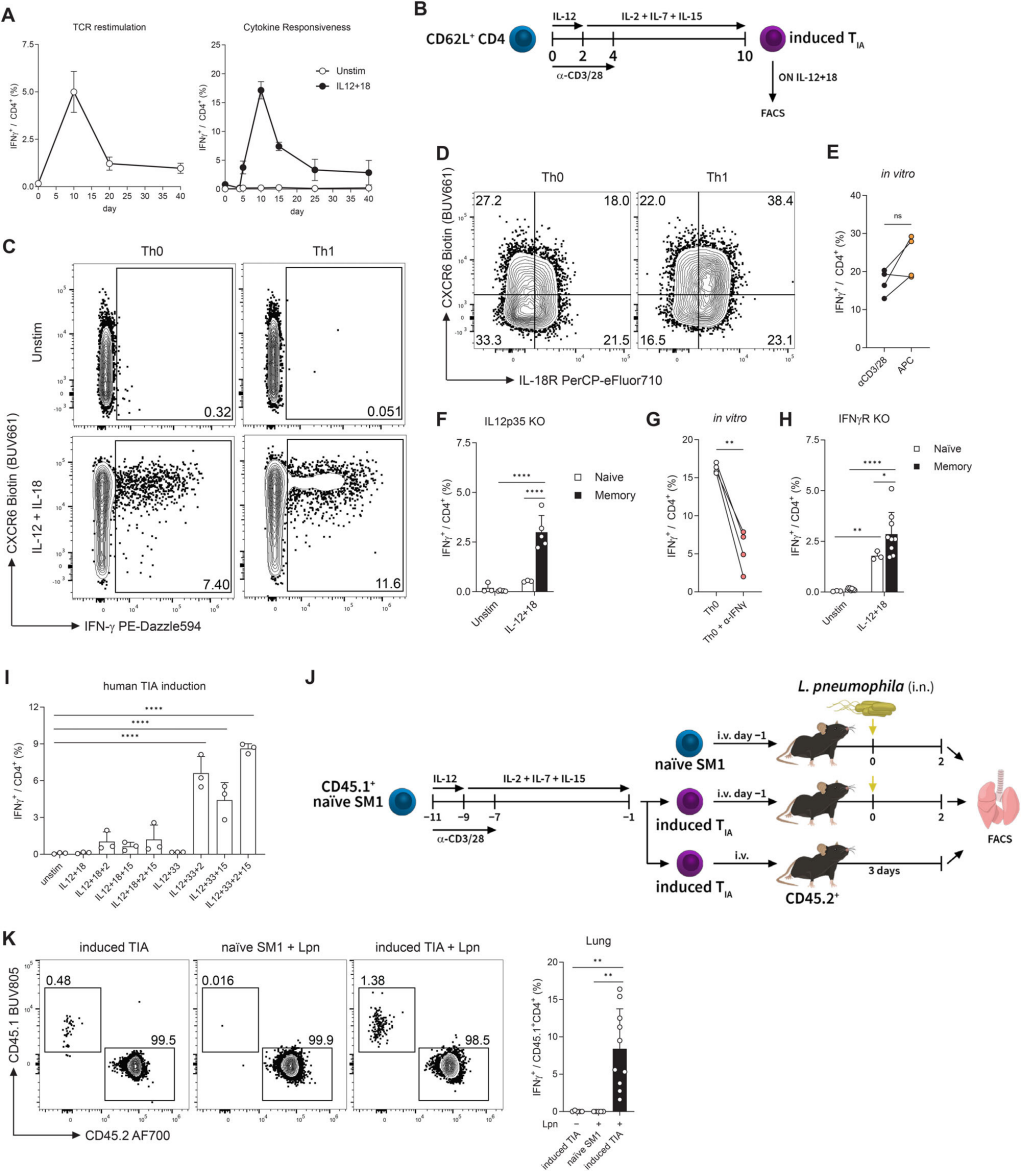
CD4 T_IA_ cells can be induced in vitro and are functional in vivo. (A) CD4 T cells of LCMV‐experienced mice were isolated at indicated time points and re‐stimulated with gp61 (left) or stimulated overnight with cytokines or left untreated (right) and analyzed for IFN‐γ production (*n* = 3–7, two independent experiments). (B) Experimental scheme of the induction protocol used in (C) and (D). Th0 cells were not polarized by IL‐12 in the first 2 days of the protocol. (C) IFN‐γ response of CD4 T cells after completing the induction protocol. (D) IL‐18R and CXCR6 expression of CD4 T cells after the 10‐Day protocol without additional stimulation. (E) Naïve SM1 CD4 T Cells in the αCD3/28 group were treated as described in (B) while those in the APC group lacked αCD3/28‐mediated stimulation but were co‐cultured with gp61‐pulsed APCs in the presence of IL‐12 for 48 h instead (*n* = 4, two independent experiments, paired *t*‐test). (F) Splenic CD4 T cells of naïve or LCMV WE memory IL‐12p35 KO mice were isolated and stimulated overnight with cytokines or left untreated (*n* = 3–5, two independent experiments, two‐way ANOVA with Šídák). (G) Naïve CD4 T cells were treated as described in (B) without the presence of IL‐12 and were left untreated or treated with neutralizing α‐IFN‐γ antibody for 4 days (*n* = 4, two independent experiments, paired *t*‐test). (H) Splenic CD4 T cells of naïve or LCMV WE memory IFNγR KO mice were stimulated overnight with cytokines or left untreated (*n* = 3–9, two independent experiments; two‐way ANOVA with Šídák). (I) Sorted CD45RA^+^CD45RO^−^ human CD4 T cells induced using the protocol depicted in (B) were stimulated with cytokines for 48 h to determine their IFN‐γ response (*n* = 3, two independent experiments, one‐way ANOVA with Dunnett). (J) Experimental setup used in (K). (K) Representative FACS plots gated on CD4 T cells (left) and IFN‐γ response of SM1 cells (right) 3 days after adoptive transfer (*n* = 5–10, two independent experiments, one‐way ANOVA with Kruskal–Wallis; Lpn: *L. pneumophila*).

Given that the cytokine responsiveness could be detected in both murine and human CD4 T cells and the diverse disease settings from which murine CD4 T_IA_ cells can originate, we sought to determine when during the infection they first appear. We infected mice with LCMV WE and isolated CD4 T cells at Days 4, 5, 10, 15, 25, and 40 of the infection, which were then stimulated with our cytokine combination to determine their ability to produce IFN‐γ in response to this TCR‐independent stimulation. To our surprise, the responsiveness kinetic of the CD4 T cells followed the classical T cell activation kinetic with a peak response at Day 10, followed by a contraction and a memory phase (Figure 3A).

Given the similarities of the kinetics of CD4 T_IA_ responsiveness and the classical T cell response, we set out to determine whether CD4 T_IA_ cells could be induced by classical T cell differentiation of naïve CD4 T cells in vitro. We sorted splenic CD62L^+^ CD4 T cells from naïve mice and stimulated them for 4 days with anti‐CD3 and anti‐CD28 antibodies followed by a 6‐day resting period (Figure 3B). IL‐12 was added during the first 2 days of the protocol to polarize cells towards Th1, while cells without IL‐12 (Th0) were used as controls. After this 10‐day protocol, cells were stimulated with IL‐12 and IL‐18 overnight or left unstimulated. Surprisingly, not only in vitro‐generated Th1 cells, but also Th0 cells could respond to cytokine stimulation and produce IFN‐γ (Figure 3C), revealing that classical activation through TCR together with co‐stimulation is sufficient to induce CD4 T_IA_ cells. Nevertheless, the Th1 polarization through addition of IL‐12 during the first 2 days of the protocol yielded a greater frequency of responding cells capable of producing IFN‐γ. In addition, the Th1 cultures contained an increased population of cells co‐expressing the CD4 T_IA_ markers IL‐18R and CXCR6 (Figure 3D). We further investigated the ability of peptide‐loaded APCs to induce CD4 T_IA_ cells in vitro. Naive CD62L^+^ CD4 Smarta (SM1) T cells, which bear a transgenic TCR specific for the LCMV gp61 peptide, were sorted and co‐cultured with gp61‐pulsed APCs in the presence of IL‐12 for the first 48 h of the 4‐day culture. Peptide‐loaded APCs were indeed capable of promoting CD4 T_IA_ cell differentiation in vitro, to a similar degree as anti‐CD3/CD28 stimulation (Figure 3E).

As IL‐12 plays a role in the polarization of Th1 cells, we explored their requirement for the generation of CD4 T_IA_ cells. Mice deficient in IL‐12 (IL12p35 KO) were infected with LCMV WE and allowed to clear the infection and develop T cell memory (at least 40 days post infection). Cytokine stimulation of CD4 T cells isolated from naïve and memory mice revealed that IL‐12 signaling is not essential for CD4 T_IA_ cell generation as CD4 T cells from memory IL12p35 KO showed a higher IFN‐γ response upon IL‐12 and IL‐18 stimulation than naïve controls (Figures 3F). In addition to IL‐12, IFN‐γ also polarizes CD4 T cells towards a Th1 fate. Due to the potential of Th0 cells to secrete IFN‐γ, we used neutralizing antibodies to inhibit IFN‐γ signaling in our induction protocol where IL‐12 was lacking (Th0). Indeed, IFN‐γ neutralization led to a partial but significant reduction in CD4 T cells capable of producing IFN‐γ upon cytokine stimulation (Figure 3G). However, LCMV‐experienced mice lacking the IFN‐γ receptor (IFNγR KO) were still able to generate CD4 T_IA_ cells (Figure 3H), highlighting that IFN‐γ is not essential for this process. Taken together, these results indicate that Th1 polarizing cytokines are not essential for the generation of CD4 T_IA_ cells but can augment the proportion of CD4 T cells that gain T_IA_ functionality.

Next, we explored whether in vitro differentiation of naïve human CD4 T cells could render them responsive to cytokine stimulation. For this, sorted CD45RA^+^CD45RO^−^ CD4 T cells were differentiated using the same induction protocol as for generating murine CD4 T_IA_ cells in vitro (Figure 3B). Indeed, after in vitro differentiation of naïve CD4 T cells, cytokine stimulation with IL‐2 and/or IL‐15 in combination with IL‐12 and IL‐33, but not IL‐12 and IL‐18, was able to elicit an IFN‐γ response (Figure 3I). Thus, similar to murine CD4 T cells, human CD4 T cells can be differentiated in vitro to become responsive to cytokine stimulation. Additionally, like sorted memory Th1 cells from healthy donors (Figure 2A), in vitro‐generated CD4 T_IA_ cells respond more strongly to IL‐33 than IL‐18 stimulation.

While the in vitro‐generated CD4 T_IA_ cells phenotypically and functionally resemble those generated in vivo, it is unclear whether these cells also have the same in vivo functionality. To test this, we induced CD4 T_IA_ cells from naïve SM1 mice, which do not recognize *L. pneumophila* [2, 11]. To this end, naïve or in vitro‐generated SM1 CD4 T_IA_ cells were transferred into congenically‐marked naïve recipient mice which were then infected intranasally with *L. pneumophila* or left naïve as controls (Figure 3J). On Day 2 of the infection, lungs were harvested and analyzed for the IFN‐γ response. Importantly, cells were only incubated with brefeldin A and not re‐stimulated, in order to determine whether a bona fide T_IA_ response is occurring. Mice that had received in vitro generated (induced) T_IA_ SM1 cells showed a higher frequency of transferred cells compared to mice that had received naïve SM1 cells (Figure 3K). While transferred cells could be detected in the lungs of both groups that received induced T_IA_ SM1 cells, an IFN‐γ response could only be observed upon *L. pneumophila* infection. This TCR‐independent response thus confirms that in vitro‐induced T_IA_ cells can indeed act in an innate manner in vivo.

Thus, classical TCR stimulation and CD28 co‐stimulation is sufficient for the in vitro generation of CD4 T_IA_ cells from naïve CD4 T cells, but it can be further enhanced by IL‐12‐mediated polarization. Importantly, in vitro‐induced CD4 T_IA_ cells are functional in vivo and respond in an innate manner upon infectious challenge.

## 3 Discussion

Unlike experimental conditions with murine models, the general population is constantly facing immunological challenges. With the capability to form memory, this interface with the environment shapes the immune system and could contribute to the increased individual variability observed with aging [12]. We have previously demonstrated how past encounters can modulate the outcome of heterologous challenges through CD4 T_IA_ cells [2]. Here we show that Th1 cells generated in diverse settings possess the potential to respond to cytokine stimulation in a TCR‐independent manner. Upon classical T cell activation, Th1 cells gain the ability to produce IFN‐γ after cytokine stimulation alone. This innate capability was also obtained by in vitro‐generated T_IA_ cells which exerted their TCR‐independent function in vivo upon *L. pneumophila* challenge.

Our previous study of CD4 T_IA_ cells has revealed IL‐18R and CXCR6 co‐expression to be a characteristic marker combination to identify CD4 T_IA_ cells [2]. Here we show that IL‐18R^+^CXCR6^+^ CD4 T cells can be found after acute or chronic LCMV infection as well as a local influenza virus infection. CD4 T cells isolated after clearance of the acute infections or during the chronic phase of the LCMV Cl13 infection demonstrated a responsiveness to IL‐12 and IL‐18 stimulation by IFN‐γ production, regardless of their respective pathogen encounter. In addition, the responsiveness is present during the acute, contraction and memory stages of an infection and can be induced in vitro by TCR engagement and co‐stimulation alone. This emergence of Th1‐responding T_IA_ cells upon various Th1‐dominated infections hints towards an ubiquitous nature of the gained innate capability. Indeed, the TCR‐independent response of CD4 T cells is not restricted to Type 1 immunity, as Th2 and Th17 cells have also been shown to respond with IL‐13 or IL‐17A, respectively, upon cytokine stimulation or heterologous challenge [4, 13, 14].

Similar to murine CD4 T_IA_ cells, human memory Th1 cells from healthy donors were able to mount an IFN‐γ response upon cytokine stimulation. While the murine response to IL‐12 and IL‐18 stimulation is greater than to IL‐12 and IL‐33 stimulation [2], the stimulation of human CD4 T cells was most effective when IL‐33 instead of IL‐18 was used in combination with IL‐12, IL‐2, and IL‐15. This was especially true for CD4 T_IA_ cells differentiated from naïve CD4 T cells in vitro.

Importantly, in vitro‐generated CD4 T_IA_ cells are functional in vivo, and produce IFN‐γ in an antigen‐independent manner during the innate stages of an unrelated infection. Given the simple requirements for naïve CD4 T cells to become receptive to cytokine stimulation, it stands to reason that CD4 T_IA_ cells are likely more ubiquitous than so far anticipated. Due to the fact that these cells functionally modulate heterologous challenges in vivo as seen in their protective effects during an infectious challenge as well as their contribution to an accelerated onset of autoimmunity [2], it seems likely that T_IA_ cells are involved in more diseases than thus far appreciated and could potentially present themselves as interesting therapeutic targets. For instance, a recent study found that a majority of CD4 T cells found in lung and colorectal tumors are likely tumor‐unspecific bystander cells [15]. Targeted delivery of cytokines to the tumor microenvironment could thus be a potent mechanism to induce effector responses in these cells. Currently, strategies are being developed to deliver cytokines such as IL‐12 and IL‐18 to the tumor microenvironment [16, 17]. However, our data presented here suggests a superior strategy to be a delivery of IL‐12 and IL‐33 combined with CD122‐biased IL‐2 [18].

In summary, our findings show that naïve CD4 T cells can acquire innate‐acting capability through classical T cell activation via TCR stimulation and CD28 co‐stimulation in vitro. Cytokine stimulation alone is sufficient to evoke an IFN‐γ response in CD4 T_IA_ cells generated in vitro as well as isolated from various murine infection models or human PBMCs, thus revealing a broad adoption of innate‐acting capability by activated or memory CD4 T cells.

## 4 Materials and Methods

### 4.1 Mice

C57BL/6 (B6) mice were purchased from Janvier Labs and IL12p35 KO (Jackson Laboratory #002692, [19]), IFNγR KO (Jackson Laboratory #003288, [20]), and Smarta [11] mice have been previously described. Animals were bred and housed in SPF facilities at LASC Zürich, Switzerland. All experiments were performed in accordance with institutional policies and regulations of the relevant animal welfare acts and have been reviewed and approved by the Cantonal veterinary office.

### 4.2 Viral and Bacterial Infections and Adoptive Cell Transfers

For the acute and chronic LCMV infections, 200 FFU LCMV WE and 2 × 10^6^ FFU LCMV Cl13, respectively, were injected i.v. Influenza A virus infections were performed with 200 PFU of influenza PR8‐gp33 virus given intranasally. For systemic *L. pneumophila* infections, 3 × 10^6^ CFU of *L. pneumophila* JR32 FlaA^−^ [21] were injected intravenously, 37 days later a booster of 3 × 10^6^ CFU (i.v.) was given and mice were analyzed at least 40 days after the booster. For local *L. pneumophila* infections, 3 × 10^6^ CFU of *L. pneumophila* JR32 FlaA^−^ was given intranasally.

For SM1 cell transfers, CD4 T cells from SM1 mice (CD45.1) were purified using MojoSort Mouse CD4 Nanobeads (BioLegend) and 2.5 × 10^5^ naïve or T_IA_‐induced SM1 cells were adoptively transferred i.v. into B6 recipient mice one day prior to local *L. pneumophila* infection.

### 4.3 In Vitro Assays

To obtain single‐cell suspensions, spleens were mechanically disrupted and red blood cells were lysed with ACK buffer (155 mM NH_4_Cl, 10 mM KHCO_3_, 0.1 mM Na_2_EDTA, pH: 7.4) for 3 min. Lungs were enzymatically digested with collagenase D (Gibco) and DNase I (VWR) for 30 min, and immune cells isolated using a 30% Percoll (GE Healthcare) gradient. Human blood was collected from healthy donors, in accordance with the approved study by the Cantonal Ethics Committee of Zurich (BASEC 2024‐01233). PBMCs were isolated using a Ficoll gradient (Cytiva).

For the isolation of CD4 T cells, cells were magnetically sorted using MojoSort Mouse CD4 Nanobeads (BioLegend) or the human CD4+ T Cell Isolation Kit (Miltenyi Biotec). Upon isolation, 2 × 10^5^ murine CD4 T cells were incubated with IL‐12 and IL‐18 (both at 10 ng/mL) for 12–18 h in U‐bottom plates or re‐stimulated for 4 h with gp61‐80 (gp61, 1 µg/mL, GLNGPDIYKGVYQFKSVEFD, EMC microcollections GmbH). Human CD4 T cells were further sorted into naïve and memory Th1 cells and 2 × 10^5^ cells were stimulated for 48 h with IL‐2 (100 U/mL), IL‐12 (10 ng/mL), IL‐15 (20 ng/mL), IL‐18 (50 ng/mL), or IL‐33 (50 ng/mL) in U‐bottom plates.

For in vitro induction of T_IA_ cells, 10^5^ naïve CD4 T cells were stimulated in flat bottom plates coated with anti‐CD3 (mouse: 2 µg/mL, clone 145‐2C11, BioXCell; human: 3 µg/mL, clone UCHT, BD Biosciences) in the presence of soluble anti‐CD28 (mouse: 2 µg/mL, clone PV‐1, BioXCell; human: 1 µg/mL, clone 28.2, BD Biosciences) for 96 h in RPMI 1640 media (Gibco) supplemented with 10% heat‐inactivated FCS, sodium pyruvate (Gibco), 200 µM L‐Glutamine (Gibco), 10 U/mL Penicillin–Streptomycin (Gibco), MEM non‐essential amino acids (Gibco), MEM vitamin solution (Gibco), and 50 µM β‐mercaptoethanol. Where indicated, cells were additionally incubated with IL‐12 (10 ng/mL) during the first 48 h or neutralizing α‐IFN‐γ antibody (10 µg/mL, XMG1.2; BioLegend) during the first 96 h of the stimulation. For co‐cultures with peptide‐loaded APCs, 10^6^ APCs were pulsed with gp61 (1 µg/mL; GLKGPDIYKGVYQFKSVEFD). From the third day onwards, cells were incubated with survival cytokines IL‐2 (mouse: 20 U/mL; human: 100 U/mL), IL‐7 (10 ng/mL), and IL‐15 (mouse: 50 ng/mL, human: 20 ng/mL). After the 96 h of stimulation, cells were switched to U‐bottom plates for the remaining 6 days of rest and subsequent cytokine stimulations. Recombinant cytokines were purchased from Biolegend with the exception of human IL‐2 (R&D Systems). All incubations were performed at 37°C and 10% CO_2_.

### 4.4 Flow Cytometry

FACS stainings were performed on single cell suspensions. When staining for intracellular cytokines, cells were incubated with brefeldin A (BioLegend) for 4 h at 37°C in 10% CO_2_ prior to staining. Fluorescently labeled antibodies were purchased from BD Bioscience, Invitrogen, and Biolegend. Zombie NIR fixable dye (Biolegend) or LIVE/DEAD Fixable Blue dye (Invitrogen) was used to exclude dead cells and debris. Cells were permeabilized and fixed using the Cytofix/Cytoperm kit (BD Biosciences).

The following antibodies were used for flow cytometry: αCD4‐BUV496 (RM4‐5), αCD44‐BUV496 (IM7), αCD8a‐BUV395 (53‐6.7), αPD‐1‐BUV737 (RMP1‐30), αCD45.1‐BUV805, αCD62L‐BUV805 (MEL‐14), αCD49d‐BV480 (R1‐2), CD103‐BB700 (2E7), αST2‐R718 (U29‐93), and Streptavidin‐BUV661 were purchased from BD. αIL‐18Ra‐eFluor450 or PerCP/eFluor710 (P3TUNYA), αIL‐18Ra‐PE (H44), ST2‐PerCP‐eFluor710 (hIL33Rcap), αKLRG1‐SB702 (2F1), αCD29‐FITC (HMb1‐1), and αNKG2D‐PE‐Cy7 (CX5) were all purchased from eBioscience. αCD4‐BV421 or APC (RM4‐5) as well as Pacific Blue (SK3), αCD45RA‐BV510 (HI100), αCX3CR1‐Pacific Blue (SA011F11), αLy‐6C‐BV510 (HK1.4), αCD44‐PerCP (IM7), αIFNγ‐PE‐Dazzle594 (XMG1.2) or PE‐Cy7 (4S.B3), αB220‐PerCP‐Cy5.5 (RA3‐6B2), αCXCR3‐BV650 or PE (CXCR3‐173) as well as BV650 (G025H7), αCD45.2‐PerCP or AF700 (104), αCD62L‐BV650 (MEL‐14), αCD25‐PE‐Cy5 (BC96) or BV605 (PC61), αPD‐1‐BV605 (EH12.2H7), αCD69‐BV785 (H1.2F3), αCCR7‐PE‐Cy7 (4B12), αCD127‐APC (S18006K), αCD85k‐AF647 (H1.1), αOX‐40‐APC‐Fire750 (OX‐86), αCXCR6‐Biotin (SA051D1 or K041E5), αNKG2D‐PE (CX5), αCD122‐PE‐Cy5 (TM‐b1), αTIGIT‐BV421 (1G9), and αCD45RO‐APC (UCHL1) were all purchased from Biolegend.

BD FACSAria III or BD FACSymphony S6 was used for cell sorting. Data was acquired on a BD LSR Fortessa, BD FACSymphony A5 analyzer (BD Biosciences), or Cytek Aurora and analyzed using SpectroFlo (Cytek) and Flowjo software (TreeStar).

### 4.5 Analysis of Flow Cytometry and Single‐Cell RNAseq Data in R

For the analysis of flow cytometry data in R, FlowJo gating was used to select the population of interest by using the packages CytoML [22], flowCore [23], and flowWorkspace [24]. Dimensionality reduction and visualization was done using the CATALYST package [25, 26].

The single‐cell RNA‐seq dataset from Travaglini et al. [10] was analyzed using the Seurat package [27]. The subset of CD4 T cells were selected based on the “free_annotation” property included in the dataset. Data was normalized, scaled and integrated using Seurat's NormalizeData, ScaleData, and IntegrateLayers functions. Dimensionality reduction was performed using the first 10 principal components and running Seurat's FindNeighbors, FindClusters, and RunUMAP with a resolution of 0.5. Data was plotted using Seurat's DimPlot, FeaturePlot, and DotPlot functions. Finally, the packages tidyverse [28], ggplot2, and qs were used for both flow cytometry and transcriptomic data.

### 4.6 Statistics

All statistical analyses were performed using GraphPad Prism and were two‐sided. Data was tested for normality with Shapiro–Wilk test and QQ‐plot analysis. All results were obtained from at least two independent experiments and are shown as mean ± SD. Statistical significance is defined as *p* < 0.05 and shown as *, *p* < 0.01 as **, *p* < 0.001 as ***, and *p* < 0.0001 as ****. *p* values between 0.10 and 0.05 are indicated by the exact value.

## Author Contributions

**Nima Yassini**: conceptualization, experimentation, analysis, formal analysis, writing – original draft. **Eva Goljat**: experimentation. **Camilla Panetti**: experimentation. **Matthias Rath**: human sample collection. **Nicole Joller**: conceptualization, writing – original draft, funding acquisition, supervision.

## Conflicts of Interest

The authors declare no conflicts of interest.

## Peer Review

The peer review history for this article is available at https://publons.com/publon/10.1002/eji.70054.

## Supporting information




**Supporting Information file 1**: eji70054‐sup‐0001‐FigureS1.pdf

## Data Availability

The data that support the findings of this study have been published previously and are available via Synapse (https://www.synapse.org/#!Synapse:syn21041850) as well as the European Genome‐phenome Archive (EGA) with the accession EGAS00001004344, or are available from the authors upon reasonable request.
