# Supplementary material for: CD4 T Cells Acquire Innate Capability Upon Classical T Cell Activation
Source: Eur J Immunol. 2025 Sep 9;55(9):e70054. doi: 10.1002/eji.70054 (PMC12419137; doi:10.1002/eji.70054)
Supplement: Supplementary file 1 — Supporting Information file 1: eji70054‐sup‐0001‐FigureS1.pdf [file EJI-55-e70054-s001.pdf]

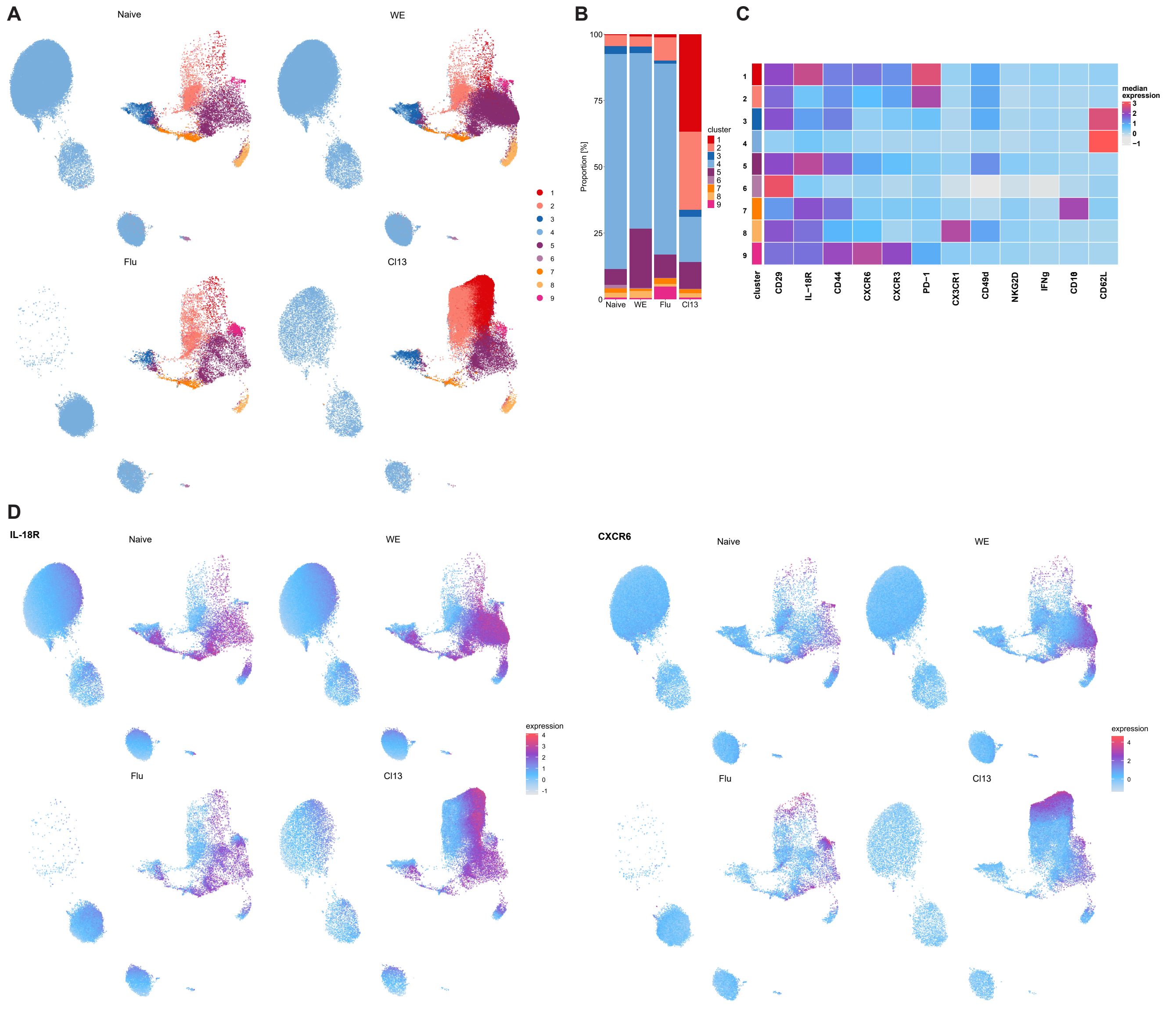

Figure S1. Marker expression among total CD4 T cells. Control and LCMV-experienced (A–D) mice were infected with LCMV WE (i.v.), LCMV Cl13 (i.v.), influenza A virus (Flu, i.n.) or left naïve and immune cells of the lung were isolated from LCMV WE or Flu infected mice (day ≥ 40) or LCMV Cl13 infected mice (day ≥ 25). CD4 T cells were analyzed using unsupervised clustering (n=2–5, two independent experiments). (A) UMAP, (B) proportion of each cluster per condition and (C) heatmap showing marker expression within these clusters. (D) Expression of IL-18R and CXCR6 shown by UMAP.
